# Chemical Profiling of Polar Lipids and the Polyphenolic Fraction of Commercial Italian *Phaseolus* Seeds by UHPLC-HRMS and Biological Evaluation

**DOI:** 10.3390/biom14101336

**Published:** 2024-10-20

**Authors:** Vadym Samukha, Francesca Fantasma, Gilda D’Urso, Ester Colarusso, Anna Schettino, Noemi Marigliano, Maria Giovanna Chini, Gabriella Saviano, Vincenzo De Felice, Gianluigi Lauro, Francesco Maione, Giuseppe Bifulco, Agostino Casapullo, Maria Iorizzi

**Affiliations:** 1 Department of Biosciences and Territory, University of Molise, Contrada Fonte Lappone, 86090 Isernia, Italy; v.samukha@studenti.unimol.it (V.S.); fantasma@unimol.it (F.F.); saviano@unimol.it (G.S.); defelice@unimol.it (V.D.F.); iorizzi@unimol.it (M.I.); 2 Department of Pharmacy, University of Salerno, Via Giovanni Paolo II 132, 84084 Fisciano, Italy; gidurso@unisa.it (G.D.); ecolarusso@unisa.it (E.C.); glauro@unisa.it (G.L.); bifulco@unisa.it (G.B.); 3 ImmunoPharmaLab, Department of Pharmacy, School of Medicine and Surgery, University of Naples Federico II, Via Domenico Montesano 49, 80131 Naples, Italy; anna.schettino2@unina.it (A.S.); noemi.marigliano@outlook.com (N.M.); francesco.maione@unina.it (F.M.)

**Keywords:** *Phaseolus vulgaris* L., food fingerprint, LC-MS/MS, polar lipids, polyphenols, nutraceuticals, minerals, anti-inflammatory effect

## Abstract

The common bean (*Phaseolus vulgaris* L.) is one of the oldest food crops in the world. In this study, the ultra-high-performance liquid chromatography high-resolution mass spectrometry (UHPLC-MS/MS) technique was used to characterize the polar lipid composition and polyphenolic fraction of five bean varieties commonly consumed in Italy: Cannellino (PVCA), Controne (PVCO), Borlotti (PVBO), Stregoni (PVST), and Vellutina (PVVE). Lipid content represents a minor fraction of the whole metabolome in dry beans, and little is known about their polar lipids, which could be potentially bioactive components. Thirty-three compounds were detected through UHPLC-MS/MS, including oxylipins, phospholipids, N-acyl glycerolipids, and several fatty acids. The dichloromethane extracts were subjected to principal component analysis (PCA), with the results showing greater differentiation for the Borlotti variety. Moreover, 27 components belonging to different polyphenol classes, such as phenolic acids, flavonoids, catechins, anthocyanins and their glycosides, and some saponins, were identified in the hydroalcoholic seed extracts. In addition, the mineral content of the beans was determined. Considering the high number of compounds in the five apolar seed extracts, all samples were examined to determine their in vitro inhibitory activity against the enzyme cyclooxygenase-2 (COX-2), which is inducible in inflammatory cells and mediates inflammatory responses. Only PVCO showed the best inhibition of the COX-2 enzyme with an IC_50_ = 31.15 ± 2.16 µg/mL. In light of these results, the potential anti-inflammatory properties of PVCO were evaluated in the LPS-stimulated murine macrophage cell line J774A.1. Herein, we demonstrate, for the first time, that PVCO at 30 µg/mL can significantly reduce the release of TNF-α, with a less significant anti-inflammatory effect being observed in terms of IL-6 release.

## 1. Introduction

The kidney bean (*Phaseolus vulgaris* L.) is one of the most important leguminous plants globally, due in part to its high nutritional content. It is a major food source of protein (20–25%) and complex carbohydrates (50–60%), and it is an excellent source of macro- and micronutrients such as dietary fiber and polyunsaturated fatty acids [1,2]. Similar to other legumes, beans are commonly included in the diets of people in various countries, especially in developing regions [1,2,3]. The results of a number of studies have shown that consumption of legumes decreases the risk of cardiovascular diseases, type 2 diabetes, stomach and prostate cancer, and obesity, among other roles [4,5,6,7]. These properties of pulses can be attributed to their bioactive compounds [8,9], such as phenolic compounds, flavonoids, phenolic acids, tannins, and triterpenic acids, among others [10]. Phenolic compounds are distributed in the seed coat and cotyledons of legumes, being mainly responsible for seed coat color depending on their composition and concentration. Hence, in general, higher phenolic content is observed in more pigmented beans [11,12], with condensed tannins, anthocyanins, and flavonoids mostly present in the seed coat, while the cotyledon is rich in phenolic acids, such as ferulic, synaptic, chlorogenic, and other hydroxycinnamic acids, which are present in lower amounts [13]. Various authors have reported that beans with colored skin, such as kidney beans or black beans, possess strong antioxidant activity or higher phenolic content, as measured using different model systems [14,15,16].

However, beans also exert negative effects on health. Even though α-galactooligosaccharides, such as stachyose, possess immunomodulatory activity in vitro, as reported by Dai et al. [17], the absence of α-galactosidase in humans induces anaerobic fermentation of the oligosaccharides by microorganisms in the large intestine, producing carbon dioxide, hydrogen, and methane, which can cause flatulence and diarrhea [18].

Consumption of raw or inadequately cooked beans is known to cause poisoning, characterized by nausea, vomiting, diarrhea, severe acute gastroenteritis, and intestinal malabsorption [19]. This toxicity has been attributed to active lectins, one of the major classes of common bean storage proteins. However, lectins reduce the levels of glucose in the blood by forming complexes with α-amylase, and they may, therefore, be considered beneficial for diabetic patients, increasing interest in exploiting the biomedical applications of these proteins [20,21]. Other antinutritional components present in beans are tannins, which, although recognized for their antioxidant capacity [22], can form complexes with minerals, reducing their bioavailability, and can interfere with digestion due to their anti-trypsin and anti-amylase activities [23,24,25].

The lipid fraction of dry common beans is roughly 2.20–5.03% and mainly comprises an acyl-glyceride mixture of mono- and polyunsaturated fatty acids, with the highest concentration of linoleic (18:2) and linolenic (18:3) acids [26,27].

Although *P. vulgaris* has been the subject of various investigations, few studies in the literature detail the content and characterization of the lipid profile of *P. vulgaris* using mass spectrometry. In 2005, Yoshida et al. reported the presence of phospholipids (PLs), such as phosphatidylcholine (PC), phosphatidylethanolamine (PE), or phosphatidylinositol (PI), identified through Thin-Layer Chromatography (TLC) [27]. Lipids can be divided into neutral and polar lipids. Neutral lipids mainly consist of triacylglycerols, wax, and terpenes. Polar lipids encompass a diverse array of biomolecules spanning several chemical categories, including phospholipids, glycolipids, and sphingolipids. They have an amphiphilic nature and play a variety of biological functions [28]. Each PL category is distinguished by its unique polar component, or ‘head group’, which, in turn, comprises numerous molecular variants. These variants are defined structurally by fatty acids or other hydrocarbon segments, which vary in both chain length and saturation level. Dietary phospholipids are believed to maintain or enhance brain function and health since they are abundant in the nervous system and have long been suspected to be involved in brain maturation and function [29]. In addition, some PLs appear to contribute directly and/or indirectly to various inflammatory cascades and are involved in the initiation, progression, and often the cure of inflammatory diseases [30].

**Figure 1:**
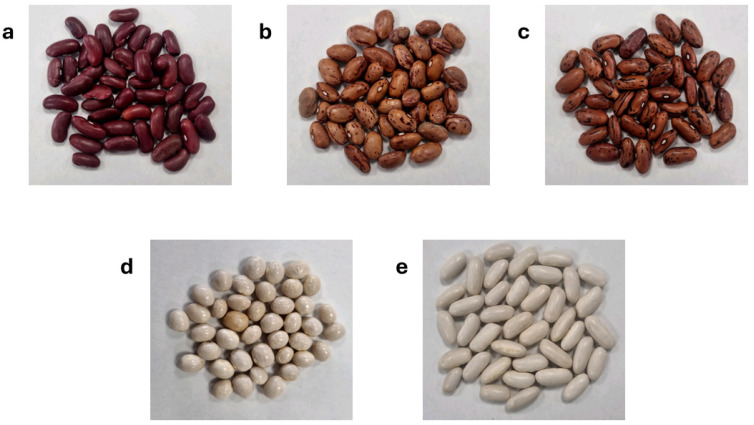
Selected Italian commercial bean varieties: Vellutina (PVVE) (**a**); Borlotti (PVBO) (**b**); Stregoni (PVST) (**c**); Controne (PVCO) (**d**); Cannellino (PVCA) (**e**).

In our study, we focused on five distinct commercial Italian cultivars of *Phaseolus vulgaris*: “Cannellino” (PVCA), “Controne” (PVCO), “Borlotti” (PVBO), “Stregoni” (PVST), and “Vellutina” (PVVE) (Figure 1). In our previous paper, the metabolic fingerprinting of PVCA, PVCO, and PVVE was investigated using nuclear magnetic resonance (NMR) [26]. However, it is difficult to investigate the polyphenolic content and lipid profile of the analyzed varieties of *P. vulgaris* using this metabolomics approach based only on the NMR technique. The aim of the present work was to perform a comprehensive characterization of the lipid content in the dichloromethane seed extracts and the composition of the polyphenolic fraction obtained from the hydroalcoholic extracts of the five commercial beans using the LC-ESI-HRMS (Liquid Chromatography–ElectroSpray Ionization–High-Resolution Mass Spectrometry) technique. Moreover, two Italian commercial varieties (PVBO and PVST) were included to better understand the lipidomic profile and other metabolites of *P. vulgaris*.

The use of untargeted LC-MS made it possible to determine the detailed fingerprinting of the polar lipids from *Phaseolus* seeds. In the present study, for the first time, 34 polar lipids belonging to oxylipins and glycerolipids, mainly phosphatidylcholine and phosphatidylethanolamine, were tentatively identified. Moreover, multivariate data analysis of the five cultivars was performed to classify and discriminate each sample. In addition, to gain a more comprehensive understanding of their biological activity, all five dichloromethane extracts (apolar extracts) were examined to determine their in vitro inhibitory activity against the enzyme cyclooxygenase-2 (COX-2), which is inducible in inflammatory cells and mediates inflammatory responses. Only PVCO appears to show the best inhibition of the COX-2 enzyme. Therefore, after assessing the possible cytotoxic effects, the PVCO sample was evaluated to determine its potential anti-inflammatory properties in the LPS-stimulated murine macrophage cell line J774A.1.

## 2. Materials and Methods

### 2.1. Standards and Reagents

Methanol (Sigma-Aldrich, Milan, Italy), Water milliQ, and HCl (Biochem chemopharma, Cosne-Cours-sur-Loire, France) were used for the extraction of polyphenols. Dichloromethane (Sigma-Aldrich, Milan, Italy) was used for the extraction of the lipophilic content. Methanol (Ultra LC ROMIL Pure Chemistry, Cambridge, UK), acetonitrile (Ultra LC ROMIL Pure Chemistry, Cambridge, UK), water (Ultra LC ROMIL Pure Chemistry, Cambridge, UK) and formic acid (ROMIL, Cambridge, UK) were used for the MS analysis.

Dimethyl sulfoxide (DMSO), fetal bovine serum (FBS), 4-(2-hydroxyethyl)-1-piperazineethanesulfonic acid (HEPES) buffer solution, lipopolysaccharides (LPS) from *Escherichia coli* (O55:B5, c.n.: L5418), and 3-(4,5-dimethyl-2-thiazolyl) 2,5-diphenyl-2H-tetrazolium bromide (MTT) were purchased from Sigma-Aldrich Co. (now under Merck, Darmstadt, Germany). Dulbecco’s modified Eagle’s medium (DMEM) was obtained from Corning. Unless otherwise stated, all other reagents were from BioCell (Milan, Italy).

### 2.2. Plant Material and Extraction Procedure

In this study, the bean seeds sampled were purchased in a specialized market in Naples, Italy. All samples were Italian commercial varieties: Cannellino bean (Umbria region), Controne bean (Campania region), Vellutina bean (Umbria region), Stregoni bean (Umbria region), and Borlotti bean (Umbria region).

Commercial bean seeds were ground in a mortar to obtain a fine powder. The lipophilic content of each commercial bean (1.5 g) was extracted in triplicate according to the extraction procedure previously reported by Samukha et al. [26], obtaining lipophilic extracts in dichloromethane that were centrifuged (3000 rpm for 30 min) at room temperature (RT) and dried using a Speed-Vac at 30 °C.

Polyphenols were extracted using 0.5 g of ground beans with 10 mL of a solution of CH_3_OH/H_2_O 7:3, adjusting the pH to 2 with HCl 2M due to the stability of polyphenols at low pH [31] for 3 h under shaking [32]. The extracts were then centrifuged at 5000 rpm for 10 min at RT. The obtained hydroalcoholic extracts were concentrated to a volume of 1 mL using a Rotavapor at 30 °C, transferred into Eppendorf tubes, and dried using a Speed-Vac at 30 °C. With the aim of enriching the polyphenol fraction, the hydroalcoholic extracts were purified by using 50 mg C18 cartridges (Sep-Pak Vac 1cc, Waters) with CH_3_CN/ H_2_O (ROMIL) 4:6 as the eluting phase, uploading 10 mg of the extracts in 500 µL of H_2_O (ROMIL)/0.1% formic acid (FA) to remove the sugars and the lipid component [33].

### 2.3. UHPLC-Q Exactive Orbitrap-HRMS System

Dichloromethane extracts (lipophilic extracts) were dissolved in MeOH/H_2_O (ROMIL) 8:2 and sonicated. After centrifugation (5000 rpm, 15 min, RT), the samples were transferred into micro-vials with a final concentration of 1 mg/mL in 1 mL.

The polyphenol extracts were solubilized in MeOH/H_2_O (ROMIL) 1:1 acidified with FA 0.1% *v*/*v*, centrifuged (5000 rpm, 15 min, RT), and transferred into micro-vials (Thermo Fisher Scientific, Bremen, Germany) with a final concentration of 1 mg/mL in 1 mL.

All samples were analyzed using a UHPLC system (Ultimate 3000) coupled to an Orbitrap Q-Exactive Classic mass spectrometer (Thermo Fisher Scientific, Bremen, Germany). Liquid chromatography (LC) was performed with a Luna Omega C18 LC column (3 µm, 150 × 2.1 mm) (Phenomenex, Torrance, CA, USA) for separation. A 5 µL full loop injection was used, and a gradient program starting from 5% to 95% of the B phase over 30 min was applied, where Phase A was H_2_O with 0.1% formic acid and Phase B was CH_3_CN with 0.1% formic acid [34,35]. The system included an electrospray ionization source operating in positive and negative ion switching modes. Full ion MS was set for each lipophilic extract, which was run in duplicate, and all ion fragmentation (data-dependent scan) was set as scan events for both lipophilic and polyphenol extracts, with the MS/MS fragmentation of the first five most intense ions in the full scan. Operation parameters for both negative and positive ion modes were as follows: FTMS scan mode with a mass range from 180 to 2000 *m*/*z* with a resolution of 70,000; spray voltage 3000; capillary temperature 275 °C; sheath and auxiliary gas flow (N_2_), 40 and 5; sweep gas 0; spray voltage 5.

### 2.4. Data Processing and Multivariate Analysis

LC-MS raw data obtained by full scan and data-dependent scan analysis of the lipophilic extracts were processed using Compound Discoverer (version 3.3.3.200, Thermo Fisher Scientific, Bremen) working with the “*Untargeted Lipidomics using Online Databases, LipidBlast in-silico library and LipidMaps database*” workflow, setting a threshold of 10^6^ and a mass tolerance of 5 ppm in “Detect Compounds” of the workflow. For the full scan data, “sample type” was set as “sample”. In this way, a new data matrix with peaks detected and integrated was generated and used for the multivariate analysis. For the data-dependent scan, “sample type” was set as “identification only”.

In the same way, LC-MS raw data of polyphenol extracts were processed using Compound Discoverer with the “Natural Product Unknown ID with Online and Local Database Searches” workflow. Finally, a tentative list of identified metabolites was obtained for both lipophilic and polyphenolic extracts.

The data matrix containing the integrated areas of the detected peaks of lipophilic extracts was imported to SIMCA P+ software 17.0 (Umetrix AB, Umea, Sweden), and an untargeted approach was performed with Principal Component Analysis (PCA) to observe differences or similarities among the selected varieties. Log transformation and the Pareto scale were used [36,37]. The first two components contributed to 53.36% of the total variance (R2 = 43.7% for Component 1; R2 = 9.66% for Component 2).

To understand the activity of lipophilic extracts, a targeted analysis was performed using partial least square discriminant analysis (PLS-DA). This approach was carried out by originating a new data matrix containing only the detected lipophilic compounds extracted from the untargeted data matrix. The first two components contributed to 75.9% of the total variance (R2 = 65.4% for Component 1; R2 = 10.5% for Component 2). Score scatter plots, loading plots, and VIP plots were generated.

### 2.5. Identification of Bioactive Compounds by LC-ESI-Q-Exactive MS^n^ Experiments

A multistep procedure combining computational compound identification (using the Compound Discoverer software platform version 3.3.3.200) and manual analysis with XCalibur software (version 2.2, Thermo Scientific, Waltham, MA, USA), assisted by literature research on *P. vulgaris*, was performed to identify lipids and phytochemicals, such as polyphenols. Different databases were available in Compound Discoverer, both internal with registered compounds and online freely accessible, such as ChemSpider, mzCloud, FoodDB, and PubChem. The data obtained from Compound Discoverer were filtered to include only compounds matched in at least two databases. These were then compared with manual analysis in XCalibur, considering a mass tolerance of <5 ppm. The Human Metabolome Database (HMDB, https://hmdb.ca/, accessed in 15 June 2024) and the KNApSAcK database (http://www.knapsackfamily.com/KNApSAcK/, accessed in 15 June 2024) were used to ascertain the occurrence of the identified metabolites in *P. vulgaris*.

### 2.6. Mineral Analysis

Briefly, in a Teflon tube, 0.500 ± 0.010 g of the bean sample was shredded. A closed microwave digestion system (ETHOS EASY, Milestone, Sorisole, Italy) was used to digest the samples using 5 mL of concentrated nitric acid (HNO_3_, TraceSELECT Ultra for ultratrace analysis, 67–69% Honeywell Fluka) and 2 mL of hydrogen peroxide (H_2_O_2_, for trace analysis 30% Merck) [38]. After digestion, the contents were filtered and transferred to a 25.00 mL volumetric flask measured with deionized water and then analyzed with an optical emission spectrometer with inductively coupled plasma (ICP-OES) (5800 ICP-OES, Agilent, Santa Clara, CA, USA). Argon (purity higher than 99.995%) was employed as the plasmogen and carrier gas. A standard solution of 100 mg/mL of calcium, iron, potassium, phosphorus, and sulfur and a standard solution of 10 mg/mL of copper, magnesium, manganese, and nickel dissolved in 10% nitric acid (HNO_3_) supplied by Supelco (Multielement standard solution 5 for ICP and Metalloid and non-metal mix for ICP), was used as the stock solution for calibration. The mineral contents of each sample were analyzed in triplicate.

### 2.7. Cell-Free Activity Assay on COX-2 Enzyme

All dichloromethane extracts from the five varieties of *Phaseolus vulgaris* were tested in single-dose triplicate mode at a final concentration of 40 μg/μL against the COX-2 enzyme using the COX-2 Inhibitor Screening Kit (Fluorometric—ab283401).

For the assay, 8 µL of test compounds (diluted with buffer at 2% DMSO), reference compound (Celecoxib, diluted with buffer at 2% DMSO), or vehicle (buffer at 2% DMSO) was pre-incubated for 10 min at 25 °C with a mixture containing 2 µL of Cofactor (200-fold diluted), 1 µL of COX Probe, 76 µL of COX Assay Buffer, and 1 µL of COX-2 enzyme. The reaction was initiated by adding 10 µL of the substrate (arachidonic acid activated with NaOH and 10-fold diluted).

The signal was detected by measuring the RFU (Relative Fluorescence Units) at Ex/Em = 535/587 nm on an EnSpire™ Multimode Plate Reader (PerkinElmer, Santa Clarita, CA, USA).

### 2.8. Anti-Inflammatory Activity of PVCO on Murine Macrophage Cell Line

#### 2.8.1. Cell Culture and Viability

The murine macrophage J774A.1 cell line was cultured in Petri culture dishes (100 × 20 mm) in Dulbecco’s modified Eagle’s medium (DMEM) supplemented with 10% FBS, 2 mM L-glutamine, 100 U/mL penicillin, 100 μg/mL streptomycin, 25 mM HEPES, and 130 μg/mL Na pyruvate in a humidified 5% carbon dioxide atmosphere at 37 °C. Cell monolayers were regularly collected by gentle scraping with a cell scraper, diluted 1:10 in fresh medium, and cultured to confluency at 37 °C. Cell viability was examined as previously described [39,40]. Specifically, using a colorimetric assay based on the MTT labeling reagent, which measures the level of mitochondrial dehydrogenase activity. J774A.1 cells (5 × 10^3^ per well) were seeded in 96-well plates, and after overnight incubation, were treated with PVCO (3–100 μg/mL). Those containing only DMSO at the highest concentration used in test wells (0.1%) were selected as an internal control. After 4 and 24 h, 10 µL of MTT solution (5 mg/mL in phosphate-buffered saline, PBS; pH 7.4) were added to each well, and the plates were incubated for 3 h at 37 °C. The medium was then removed, and the obtained formazan crystals were dissolved in 150 µL of DMSO for 15 min. The spectrophotometric absorbance was measured using a microtiter enzyme-linked immunosorbent assay reader (Multiskan™ GO Microplate Spectrophotometer; Thermo Scientific™) at 540 nm. The percentage of cell viability was determined by the following formula: OD of treated cells/OD of control × 100.

#### 2.8.2. Anti-Inflammatory Activity on Murine Macrophages Cell Line

The murine macrophage cell line J774A.1 was cultured as previously described [41]. Cells were seeded in Petri culture dishes (100 mm × 20 mm) at a density of 5 × 10^5^ cells per dish and allowed to grow for 24 h. The medium was then replaced, and cells were treated with LPS (10 μg/mL) in the presence or absence of the highest, but not cytotoxic, concentration of PVCO (30 μg/mL). Following incubation for 4 and 24 h, cells were collected with a cell scraper, centrifuged at 12,000 rpm for 5 min at 4 °C, and the supernatants were collected and stored at −80 °C for IL-6 and TNF-α enzyme-linked immunosorbent assay (ELISA) analysis [42].

#### 2.8.3. ELISA Assay

ELISA for TNF-α (Cat. no: 860.040.192; Diaclone) and IL-6 (Cat. no: DY406-05; R&D Systems) was carried out on supernatants from the murine macrophage J774A.1 cell line under different experimental conditions. Specifically, TNF-α and IL-6 levels were quantified by commercially available kits according to the standardized procedure described by Saviano A., Schettino A., and Coll. [42]. Briefly, 100 μL of supernatants, diluted standards, quality controls, and dilution buffer (blank) were applied on a pre-coated plate with the monoclonal antibody for 2 h. After washing, 100 μL of biotin-labelled antibody was added, and incubation continued for 1 h. The plate was washed, and 100 μL of the streptavidin-HRP conjugate was added. The plate was then incubated for a further 30 min in the dark. The addition of 100 μL of the substrate and stop solution represented the final steps before the reading of absorbance (measured at 450 nm) on a microplate reader (Multiskan^™^ GO Microplate Spectrophotometer; Thermo Scientific^™^). Antigen levels in the samples were determined using a standard curve, normalized to supernatant levels, and expressed as pg/mL [42].

## 3. Results and Discussion

### 3.1. Identification of Polar Lipids in Five Italian Cultivars of P. vulgaris Through LC-ESI-FT-MS Analysis

Our study focused on five distinct Italian cultivars of *Phaseolus vulgaris*: “Cannellino” (PVCA), “Controne” (PVCO), “Borlotti” (PVBO), “Stregoni” (PVST), and “Vellutina” (PVVE). We conducted a comprehensive analysis of the polar lipid composition present in the dichloromethane seed extracts using the LC-ESI-FT-MS technique. This MS-based metabolomic approach allowed us to explore the polar lipid profiles in detail, uncovering potential variations and unique features among the different cultivars. We aimed to gain a deeper understanding of lipid metabolism in *P. vulgaris* and provide a foundation for further research in this area. The analysis was performed in both positive and negative ionization modes to identify all forms of polar lipids (LC-MS profiles in negative and positive ion modes are reported in Supplementary Figures S1 and S2).

With this approach, we detected and identified different components, including oxylipins, phosphatidylinositol (PI) and its lyso-form (L-PI), phosphatidylethanolamine (PE) and its lyso-form (L-PE), phosphatidylcholine (PC) and its lyso-form (Lyso-PC), and N-acyl glycerophosphatidylethanolamines (NA-GPE), all listed in Table 1 and Table 2 (the fragmentation scheme of the main lipid identified is reported in Figure S8).

#### 3.1.1. Polyunsaturated Fatty Acids (PUFAs) and Oxylipins Identified in *P. vulgaris*

Polyunsaturated fatty acids (PUFAs) are essential nutrients with cardioprotective and anti-inflammatory activities, and their occurrence has already been reported in *P. vulgaris*, with linoleic acid (methyl ester) and arachidic acid as the main constituents [43,44]. Oxylipins are oxygenated polyunsaturated fatty acids that differ from each other in terms of unsaturation degree and the number of hydroxyl groups [45]. Mass spectrometry is widely regarded as an excellent analytical technique for identifying oxylipins. It not only facilitates the identification of these molecules but also allows for the precise determination of the positions of hydroxyl groups and double bonds within the molecule [46,47,48]. A detailed study of the LC-MS profiles of lipophilic extracts revealed the occurrence of compounds **1**, **2**, **6**, **8**, **10**, **20**, and **22**, which showed molecular formulas and MS/MS fragmentation patterns similar to oxylipins, with diagnostic fragments. Compounds **1** and **2** exhibited an [M − H]^−^ ion that differed by 2 Da and had molecular formulas differing by two hydrogens, suggesting a difference in unsaturation. They were identified as 9,10,13-trihydroxy-11-octadecenoic acid (9,10,13-TriHODE) and 9,12,13-trihydroxyoctadecamonoenoic acid (9,12,13-TriHOME), respectively. These identified oxylipins were detected in all varieties. To our knowledge, this is the first study documenting the presence of these compounds in *P. vulgaris* species. TriHOMEs have previously only been characterized in potato leaves [49] and onion bulbs [50].

**Table 1:** Polar lipids identified in *P. vulgaris* varieties through LC-MS analysis in negative ion mode.

N°	Compound	Rt (min)	Molecular Formula	[M − H]^−^	ppm	MS/MS
**1**	9,10,13-TriHODE	13.05	C_18_H_32_O_5_	327.2163	0.02	211.13/291.20/309.21/269.17/183.14/141.18
**2**	9,12,13-TriHOME (10)	13.72	C_18_H_34_O_5_	329.2320	−0.12	229.14/311.22/293.21/211.13/171.10
**3**	10,16-Dihydroxyhexadecanoic acid	14.75	C_16_H_32_O_4_	287.2217	0.29	269.21/141.18/189.98/109.03
**4**	11-methyl dodecadienoic acid	17.05	C_13_H_22_O_2_	209.1538	−0.97	165.16515
**5**	L-PI (18:3)	17.51	C_27_H_47_O_12_P	593.2719	−0.38	315.04/**241.01**/277.21
**6**	9,10-DiHODE	18.37	C_18_H_32_O_4_	311.2222	1.65	201.11/275.20/293.21/**171.10**
**7**	L-PE (18:3)	18.50	C_23_H_42_NO_7_P	474.2620	0.36	277.21/**214.05**/**196.04**
**8**	15,16-DiHODE	18.52	C_18_H_32_O_4_	311.2221	0.55	223.17/235.17/275.20/293.21
**9**	PE(18:1(9Z)/0:0)	19.12	C_23_H_46_NO_7_P	478.2928	0.01	281.24/**214.05**/**196.04**
**10**	9,11-Linoleic acid	19.78	C_18_H_32_O_2_	279.2323	1.69	183.70/112.9
**11**	L-PE (18:2)	19.78	C_23_H_44_NO_7_P	476.2776	0.36	279.23/**214.05**/**196.04**
**12**	OKHdiA-PE	20.15	C_30_H_52_NO_11_P	632.3192	−0.29	**279.23**/255.23/112.98/
**13**	PE(16:0/0:0)	20.19	C_21_H_44_NO_7_P	452.2769	−0.47	255.23/**214.05**/**196.04**
**14**	PI (16:0_18:3)	20.40	C_43_H_77_O_13_P	831.5008	−1.14	770.57/553.28/391.22/277.22/255.23/**241.01**/297.04/
**15**	L-PE (16:0)	20.63	C_21_H_44_NO_7_P	452.2776	0.31	255.23/**214.05**/**196.04**
**16**	Palmitic acid	20.63	C_16_H_32_O_2_	255.2322	1.61	214.07/187.06/145.02/112.98
**17**	PE(18:0/0:0)	20.70	C_23_H_48_NO_7_P	480.3084	0.13	255.23/224.07/**214.05**/**196.04**/153.00
**18**	PE(16:0/18:3(9Z,12Z,15Z))	20.99	C_39_H_72_NO_8_P	712.4914	0.39	277.22/255.23/452.28/**214.05**/**196.04**
**19**	PHHdiA-PE	21.13	C_28_H_52_NO_11_P	608.3195	0.15	255.23/313.61/401.61/112.98
**20**	(6Z)-Octadecenoicacid	21.47	C_18_H_34_O_2_	281.2479	1.61	106.04/171.07/212.09
**21**	L-PE (18:1)	21.47	C_23_H_46_NO_7_P	478.2930	0.51	281.24/**214.05**/**196.04**
**22**	(9Z)-(13S)-12_13-Epoxyoctadeca-9,11-dienoic acid	22.14	C_18_H_30_O_3_	293.2116	−1.25	236.10551; 221.15480
**23**	PE (16:0, 18:2)	22.84	C_39_H_74_NO_8_P	714.5062	−0.75	452.27/279.23/ 255.23/**214.05**/**196.04**

Phosphatidylethanolamine (PE); lyso-forms of phosphatidylethanolamine (L-PE); phosphatidylinositol (PI); lyso-forms of phosphatidylinositol (L-PI); in bold, diagnostic product ions. The compounds were identified using the “*Compound Discoverer software* version 3.3.3.200” platform and literature data, such as references [45,46,47,48].

**Table 2:** Polar lipids identified in *P. vulgaris* varieties through LC-MS analysis in positive ion mode.

N°	Compound	Rt (min)	Molecular Formula	[M + H]^+^	ppm	MS/MS
**24**	L-PC (18:3)	22.62	C_26_H_48_O_7_NP	518.3230	−2.15	**184.07**/469.80/335.25/268.89
**25**	L-PC (18:3)	22.68	C_26_H_48_O_7_NP	518.3230	−2.15	**184.07**/104.11
**26**	L-PC (18:2)	23.64	C_26_H_50_O_7_NP	520.3368	−1.72	**184.07**
**27**	L-PC (18:2)	23.98	C_26_H_50_O_7_NP	520.3368	−1.72	**184.07**
**28**	L-PC(16:0)	24.49	C_24_H_50_O_7_NP	496.3389	1.56	**184.07**/104.11/313.27
**29**	L-PC(16:0)	24.88	C_24_H_50_O_7_NP	496.3389	1.56	**184.07**/104.11/313.27
**30**	L-PC(18:1)	25.32	C_26_H_52_O_7_NP	522.3550	1.85	**184.07**
**31**	L-PC(18:1)	25.68	C_26_H_52_O_7_NP	522.3550	1.85	**184.07**/104.11
**32**	NA-GPE	22.95	C_23_H_44_O_7_NP	478.2925	−0.57	337.27/**306.28**
**33**	NA-GPE	23.30	C_23_H_44_O_7_NP	478.2925	−0.57	337.27/**306.28**/155.01

Lyso-forms of phosphatidylcholine (L-PC), N-acyl glycerophosphatidylethanolamines (NA-GPE). In bold, diagnostic product ions. The compounds were identified using the “*Compound Discoverer software* version 3.3.3.200” platform and literature data, such as references [46].

#### 3.1.2. Polar Glycerolipids

Glycerolipids can be classified based on their structure into phosphatidylcholine (PC), where the head group is choline; phosphatidylethanolamine (PE), with the ethanolamine head group; and phosphatidylinositol (PI), where the head group is inositol [51]. Phosphatidylethanolamines (PEs) and phosphatidylcholines (PCs) are the main constituents of phospholipids in food [4]. Dietary PLs are ingested as part of a normal diet and appear to bring health benefits due to their anti-inflammatory, antioxidant, anti-fibrogenic, anti-apoptotic, membrane-protective, and lipid-regulating effects, with a positive impact on various diseases, supposedly without serious side effects [30].

Phosphatidylinositol (PI) and its lyso-form (L-PI) differ in their chemical structures and biological functions: PI consists of a glycerol backbone linked to two fatty acids and a phosphorylated inositol head group. L-PI lacks one of the fatty acid chains, resulting in a single fatty acid chain attached to the glycerol backbone together with the phosphorylated inositol head group.

Phosphatidylinositol serves as a precursor for important signaling molecules, such as phosphoinositides, which play crucial roles in various cellular processes, including signal transduction, membrane trafficking, and cytoskeletal organization [52].

L-PI also participates in signaling pathways, but its specific functions may differ from those of PI. Due to its altered structure, it may have distinct interactions with enzymes or proteins.

Compounds **5** and **14** showed a molecular formula and diagnostic fragmentation pattern typical of lyso-phosphatidyl- and phosphatidylinositol, with the product ions at *m*/*z* 241 corresponding to the polar head group with the molecular formula [C_9_H_14_PO_9_]^−^ [53]. They were detected for the first time in *P. vulgaris* species and were present in all varieties.

The molecular formulae of compounds **7**, **9**, **11**–**13**, **15**, **17**–**19**, **21**, and **23** were characterized by the presence of NO_7_P heteroatoms. The MS/MS spectra exhibited diagnostic product ions at *m*/*z* 214.05 and 196.04, corresponding to deprotonated species of glycerophosphatidylethanolamine and its mono-dehydrated form, respectively. Consequently, these compounds were identified as phosphatidylethanolamine. They were detected for the first time in *P. vulgaris* species. All of these identified PEs were found in all the analyzed varieties except for compounds **18** and **23**, which were not present in the PVBO variety. Compounds **32** and **33** were detected in positive ion mode, showing the same molecular formula and MS/MS spectra but different retention times. In the MS/MS spectra, the principal product ion was also detected at *m*/*z* 306.28 with a loss of 172 Da, corresponding to the neutral loss of glycerophosphoric acid. Compounds **32** and **33**, with the same molecular formula, were putatively identified as N-acyl glycerophosphatidylethanolamines (NA-GPE).

Compounds **24**–**29** were observed in positive ion mode, showing the same MS/MS fragmentation pattern, with an [M − H]^−^ ion at *m*/*z* 184.07 belonging to the phosphocholine unit. These compounds were identified as the lyso-forms of phosphatidylcholine (L-PC), differing from each other in the presence of fatty acids.

#### 3.1.3. Other Fatty Acids

Compounds **3** and **16** were identified as saturated fatty acids, as 10, 16 dihydroxy hexadecanoic acid and palmitic acid, respectively. The latter has already been reported in *P. vulgaris*. Compound **4** was identified as 11-methyl dodecadienoic acid.

### 3.2. Multivariate Analysis of Lipophilic Extracts

**Figure 2:**
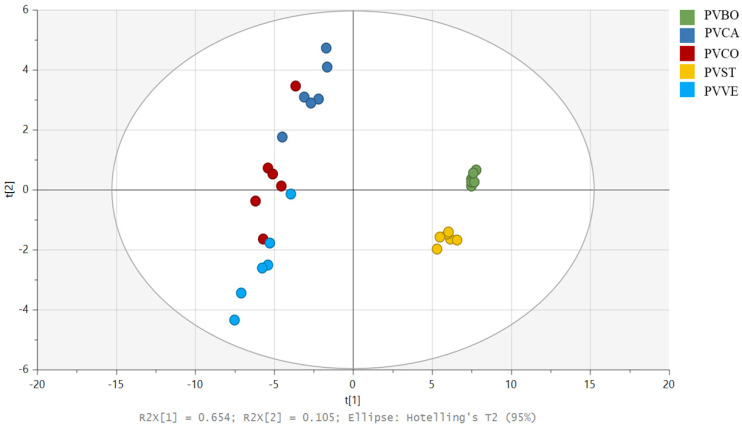
Score scatter plot of the partial least squares–discriminant analysis (PLS-DA) performed as a targeted approach on the detected compounds of the lipophilic extracts of five Italian commercial *P. vulgaris* varieties: Borlotti (PVBO) (green), Cannellino (PVCA) (blue), Controne (PVCO) (red), Stregoni (PVST) (yellow), and Vellutina (PVVE) (light blue).

**Figure 3:**
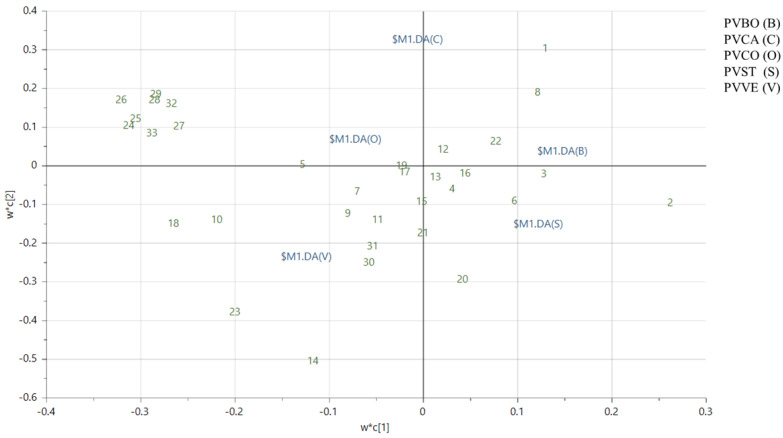
Loading scatter plot of the targeted partial least squares–discriminant analysis (PLS-DA) performed on the lipophilic extracts of five Italian commercial *P. vulgaris* varieties: Borlotti (PVBO (B)), Cannellino (PVCA (C)), Controne (PVCO (O)), Stregoni (PVST (S)), and Vellutina (PVVE (V)).

LC-MS raw data from dichloromethane extracts were processed using Compound Discoverer (CD) to obtain a dataset with integrated peak areas. The data matrix obtained with CD was then imported into SIMCA P+ software version 17.0, where an untargeted Principal Component Analysis (PCA) was performed to observe differences among the lipophilic extracts of the selected commercial beans. Figure S5 shows the PCA score scatter plot, which reveals the separation of five distinct clusters. The Borlotti variety (PVBO), represented in green on the left side of the plot, is notably different in its lipophilic content. Conversely, PVVE appears to have a similar lipophilic composition to PVCO and PVCA; in comparison, PVST shows slight differences compared to PVCA. To better classify and discriminate the polar lipids that characterize the different varieties, a targeted approach was carried out using a new data matrix containing only the detected compounds. The Partial Least-Squares–Discriminant Analysis (PLS-DA) score scatter plot (Figure 2) revealed results similar to those of the PCA, showing the separation of five distinct clusters. Despite this, the loading plot and VIP plots in our targeted analysis were more informative (Figure 3 and Figure S6a,b). Compounds **1**–**3**, **6**, **8**, and **22** were mainly present in Borlotti and Stregoni varieties. In fact, in the loading plot, they are located on the right side, corresponding to the distribution of the Borlotti and Stregoni samples in the score scatter plot. Compounds **10**, **14**, **18**, **23**–**29**, **32,** and **33** mainly characterize the Controne, Vellutina, and Cannellino varieties, and they are located on the left side of the loading plot (the bar chart showing the peak areas of polar lipids identified in negative and positive ion mode is reported in Figure S7).

### 3.3. Elemental Profiles of Seeds

The common bean is a good source of calcium, iron, magnesium, zinc, phosphorus, and selenium [15,54]. In this study, essential dietary minerals were identified, as they provide vital functions for the body [55]. The bean samples analyzed showed wide variability in the macro- and micronutrients evaluated. K, P, S, Mg, and Ca are the main cations detected in the samples, and their accumulation in the beans is influenced by environmental and genetic factors [56]. Among the micronutrients, Fe, Cu, and Mn are highly valued as their deficiency in humans can induce various chronic diseases [54]. In general, among the minerals identified in the samples, copper presented lower concentrations (from 6.53 to 9.77 mg/Kg), whereas potassium had the highest content in the samples (from 13,504.24 to 15,760.73 mg/Kg), followed by calcium (from 1013.97 to 1441.97 mg/Kg) and magnesium (from 1341.81 to 1679.2 mg/Kg).

As shown in Table 3, the nickel concentration (19.71 ± 0.01 mg/Kg) was higher in PVBO than in the other varieties and was also high when compared to the value observed in other previously studied legumes [54]. Due to its toxicity, nickel levels in food products must be continuously monitored [57].

### 3.4. Unveiling the Metabolites in the Hydroalcoholic Extracts of P. vulgaris Varieties Through LC-ESI-HRMS Analysis

LC-ESI-HRMS analysis of the hydroalcoholic extracts of the selected commercial bean varieties revealed a fascinating array of different polyphenols, primarily from the flavan-3-ol, flavonol, and flavanone classes, along with other compounds such as amino acids, phenolic acids, and saponins. This comprehensive analysis, utilizing accurate mass and MS/MS fragmentation, in conjunction with databases such as Knapsack, software for metabolite identification such as Compound Discoverer version 3.3.3.200, and our extensive literature research, led to the tentative identification of 27 metabolites (as reported in Table 4).

Our analysis was conducted in both positive and negative ion modes, with the negative ion mode producing more interesting results than the positive ion mode (LC-MS profiles in negative and positive ion modes are reported in Supplementary Figures S3 and S4).

The PVVE variety, which exhibited a more intense red coloration than the other varieties, comprised the highest number of detected compounds. Among the detected compounds in the Vellutina variety, it was interesting to note the presence of compounds **40**–**43**, corresponding to quercetin glycoside derivatives such as quercetin xylopyranosyl rutinoside, quercetin sambubioside, rutin, and quercetin hexoside, respectively. Compound **52**, detected only in PVVE at a retention time of 14.65, showed the same nominal mass as compound **53**, detected in all of the varieties at a retention time of 16.53. Owing to the high resolution of our technique, it was possible to observe that compound **52** showed an [M − H]^−^ ion at *m*/*z* 301.0724 corresponding to the molecular formula C_16_H_14_O_6_; in comparison, compound **53** showed an [M − H]^−^ ion at *m*/*z* 301.0361 corresponding to the molecular formula C_15_H_10_O_7_. The MS/MS fragmentation allowed us to identify compound **52** as hesperetin, corresponding to the flavanone eriodictyol methoxy derivative; in comparison, compound **53** was identified as quercetin. These metabolites have already been reported in *P. vulgaris* species [58]. In addition, positive ion mode revealed the presence of two anthocyanins in the PVVE variety, identified as delphinidin-hexoside (**59**) and cyanidin-hexoside (**60**), respectively. These metabolites have already been reported in red kidney beans [59,60].

Other metabolites, such as **34**, **37**, **39**, and **49**, were only detected in PVST, PVVE, and PVBO and were tentatively identified as catechin-hexoside, catechin, eriodictyol-hexoside, and kaempferol-hexoside, respectively. All of these metabolites have already been reported in beans [1].

Compound **50** was detected in PVVE, PVST, PVBO, and PVCO but not in PVCA. This compound was identified as taxifolin, also known as dihydroquercetin.

Among the other compounds, different classes of saponins were observed in each variety and were tentatively identified as soya-saponin I, soya-saponin V, sandosaponin (A/B), and dehydrosoyasaponin I, respectively, whose occurrence has already been reported in the Knapsack database for *P. vulgaris* species.

### 3.5. Biochemical Evaluation of the COX-2 Enzyme

Arachidonic acid (AA), a polyunsaturated omega-6 fatty acid, is a vital component of cell membranes and serves as a precursor to numerous bioactive lipid mediators. Through the cyclooxygenase (COX) pathway, arachidonic acid is metabolized into prostaglandins, prostacyclin, and thromboxanes, which are key players in immune response, inflammation, and homeostasis. This process is facilitated by two enzyme isoforms, cyclooxygenase-1 (COX-1) and cyclooxygenase-2 (COX-2). COX-1 is constitutively expressed and regulates normal organ function; in comparison, COX-2 is inducible in inflammatory cells and mediates inflammatory responses [61].

Due to the significant need for selective COX-2 inhibitors for anti-inflammatory purposes and considering the high lipid composition of the dichloromethane seed extracts from five varieties of *P. vulgaris*, we decided to evaluate these species to determine their potential to inhibit COX-2 enzymes.

As expected, all dichloromethane extracts were able to inhibit the COX-2 enzyme when tested at 40 µg/mL, with the only exception being PVVE (inhibition percentage = 19.6 ± 3.9%). The PVCA variant showed a weak difference compared to the other variants (inhibition percentage = 38.1 ± 7.5%).

In light of these results, we decided to investigate the IC_50_ (half-maximal inhibitory concentration) value of the PVCO, PVBO, and PVST variants. PVCO appeared to exhibit the best inhibition of the COX-2 enzyme (IC_50_ = 31.15 ± 2.16 µg/mL); in comparison, PVBO and PVST showed similar behaviour with IC_50_ values of 42.29 ± 3.11 and 43.92 ± 1.86, respectively (see Table 5 and Figure S9).

These results may be attributed to the occurrence of oxylipins in Borlotti and Stregoni varieties, such as compounds (**1**, **8**, **3**, **6,** and **22**, *vide supra*), which have already been reported in the literature to show anti-inflammatory activity [62]. Conversely, the Controne variety, located in the middle of the score scatter plot (indicating that it contains all of the polar lipids identified), is mainly characterized by the occurrence of compounds **5** and **7** (*vide supra*), such as L-PI (18:3) and L-PE (18:3).

### 3.6. Unveiling the Safety Profile and Anti-Inflammatory Activity of PVCO on the Murine Macrophage Cell Line J774A.1

**Figure 4:**
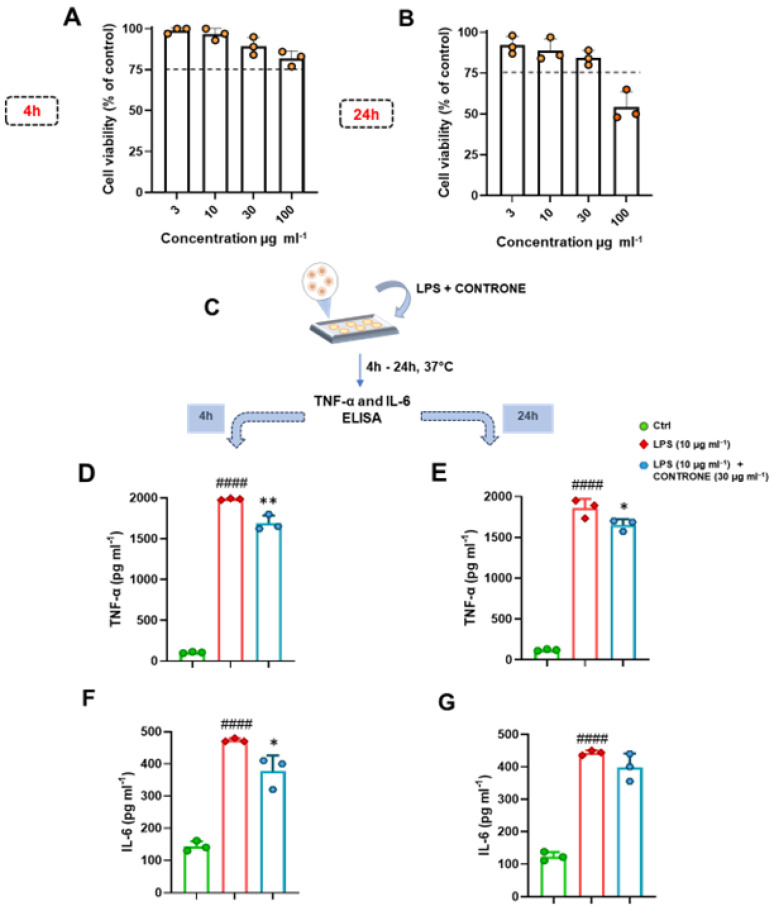
(**A**,**B**) An in vitro cytotoxic examination, evaluated using an MTT assay, was performed on the murine macrophage J774A.1 cell line, following 4 (**A**) and 24 h (**B**) of treatment with selected concentrations of PVCO (3, 10, 30, and 100 μg/mL). The dotted lines indicate the 75% cell viability limit. Data are expressed as cell viability (% of control) and presented as the means ± S.D. of three independent experiments. Our statistical analysis was conducted using one-way ANOVA followed by Bonferroni correction for multiple comparisons. (**C**,**F**) Murine macrophages J774A.1 were stimulated with LPS (10 µg/mL) and treated with PVCO at the concentration of 30 µg/mL for 4 and 24 h (**C**). Thereafter, cell supernatants were assayed using ELISA to determine TNF-α and IL-6 levels (expressed as pg/mL) at both 4 (**D**,**F**, respectively) and 24 h (**E**,**G**, respectively). Data are presented as the means ± S.D. of three independent experiments. ^####^ *p* ≤ 0.0001 vs. the Ctrl group; * *p* ≤ 0.05, ** *p* ≤ 0.01 vs. the LPS group.

Successive experiments were conducted to evaluate the potential cytotoxic effect of PVCO, in a concentration range from 3 to 100 µg/mL, on the murine macrophage cell line using an MTT assay. As shown in Figure 4, the extract displayed a safe profile up to the concentration of 30 µg/mL at both 4 (Figure 4A) and 24 h (Figure 4B). These results prompted us to test the potential anti-inflammatory profile of PVCO at the concentration of 30 µg/mL on the murine macrophage cell line. To this end, J774A.1. cells were stimulated with LPS (10 µg/mL) (Figure 4C), a TLR4 agonist that significantly enhances the induction of the inflammatory reaction in monocytes/macrophages [63]. As shown in Figure 4D–G, co-treatment of the PVCO sample at the concentration of 30 µg/mL significantly reduced the release of TNF-α at both 4 (Figure 4D) and 24 h (Figure 4E). A less significant anti-inflammatory effect was observed in terms of IL-6 release at the indicated time points (Figure 4F,G). This specific effect may be attributed to the mechanism of action of this natural extract, which requires further biological and pharmacological investigation in the future.

## 4. Conclusions

The common bean (*Phaseolus vulgaris*) is a rich source of nutritional components that make it a functional food due to its high prebiotic and nutraceutical content, which can help people maintain good health. In the present study, we analyzed the lipophilic extracts and polyphenolic profile of the seeds of five Italian commercial varieties of *P. vulgaris* using UPLC-MS/MS. To our knowledge, this is the first time a lipophilic profile has been outlined in common bean seeds, including oxylipins, phospholipids (PL), N-acyl glycerolipids, and several fatty acids. All components in the lipophilic extract were observed in the varieties examined. Moreover, the results of our untargeted and targeted multivariate analyses involving PCA and PLS-DA showed major differentiation between the Borlotti (PVBO) and Stregoni (PVST) varieties. In particular, the loading plot of our targeted analysis suggested major oxylipin content in these samples. The polyphenolic components identified in the five varieties have already been reported in *P. vulgaris* beans based on literature data. The different polyphenols are found in both free and glycosylated forms and are more abundant in beans with colored skin, whereas phenolic acids were identified in all varieties. The beans showed good mineral content, with K, P, S, Mg, and Ca comprising the main cations detected; in comparison, Fe, Cu, Mn, and Ni were highlighted as micronutrients. The five apolar seed extracts were examined to determine their in vitro inhibitory activity against the enzyme cyclooxygenase-2 (COX-2), which is inducible in inflammatory cells and mediates inflammatory responses. Only PVCO showed the best inhibition of the COX-2 enzyme with an IC_50_ = 31.15 ± 2.16 µg/mL. To test its potential anti-inflammatory profile, PVCO was evaluated in the LPS-stimulated murine macrophage cell line J774A.1. Herein, we demonstrate, for the first time, that PVCO at 30 µg/mL can significantly reduce the release of TNF-α at both 4 and 24 h. A less significant anti-inflammatory effect was observed in terms of IL-6 release at the indicated time points. Dietary PLs are ingested as part of a normal diet and appear to provide health benefits due to their anti-inflammatory activity against chronic diseases. Therefore, in light of the results obtained, PLs are potentially suitable as dietary supplements. The LC-MS technique is a powerful tool for studying metabolites that cannot be easily detected using NMR in a metabolomic approach, such as lipids and polyphenols. By combining LC-MS and NMR techniques, it is possible to obtain a complete fingerprint of selected varieties, enhance biodiversity, protect regional foods, and provide indications of geographical origin.

## Figures and Tables

**Table 3:** Concentration of minerals in the five bean varieties.

Elements	Bean Varieties				
	PVBO	PVCA	PVCO	PVST	PVVE
Ca (λ _393.366 nm_)	1013.97 ± 0.36	1114.75 ± 0.09	1441.97 ± 0.58	1083.33 ± 0.16	1127.33 ± 0.04
Cu (λ _327.395 nm_)	7.88 ± 0.01	9.77 ± 0.01	8.22 ± 0.01	7.06 ± 0.01	6.53 ± 0.01
Fe (λ _259.940 nm_)	64.39 ± 0.01	55.66 ± 0.01	46.42 ± 0.01	59.79 ± 0.01	59.24 ± 0.01
K (λ _766.491 nm_)	13,504.24 ± 0.16	15,143.07 ± 0.19	13,886.36 ± 0.49	15,073.92 ± 0.82	15,760.73 ± 2.50
Mg (λ _280.270 nm_)	1548.22 ± 0.37	1679.20 ± 0.05	1600.58 ± 0.12	1341.81 ± 0.31	1455.22 ± 0.15
Mn (λ _257.610 nm_)	15.29 ± 0.01	20.02 ± 0.01	10.64 ± 0.01	11.77 ± 0.01	15.39 ± 0.01
Ni (λ _216.555 nm_)	19.71 ± 0.01	1.46 ± 0.01	0.48 ± 0.01	0.47 ± 0.01	0.93 ± 0.01
P (λ _213.618 nm_)	4724.68 ± 0.69	5095.70 ± 0.74	3581.24 ± 0.60	4450.56 ± 0.46	5031.72 ± 0.32
S (λ _181.972 nm_)	2090.96 ± 0.18	1805.66 ± 0.33	1893.62 ± 0.11	1931.73 ± 0.45	2247.67 ± 0.20

Bean varieties: Borlotti (PVBO); Cannellino (PVCA); Controne (PVCO); Stregoni (PVST); Vellutina (PVVE). Mean ± standard deviation (mg/Kg).

**Table 4:** Metabolites identified in hydroalcoholic extracts of *P. vulgaris* varieties through LC-MS analysis in negative and positive ion mode.

**N°**	**Compound**	**Rt (min)**	**Molecular Formula**	**[M − H]^−^**	**ppm**	**MS/MS**	**Detection**
**34**	(epi)catechin-hexoside	7.77; 8.26	C_21_H_24_O_11_	451.1249	3.28	289.07 245.08 125.02	PVVE PVST PVBO
**35**	L-glutamyl-L-leucine	8.70	C_11_H_20_O_5_N_2_	259.1302	5.5	128.03 130.09	All
**36**	feruloylglucaric acid derivative	8.87	C_16_H_18_O_11_	385.0783	4.60	85.03 191.02 209.03	All
**37**	catechin/(epi-)	10.05; 10.24	C_15_H_14_O_6_	289.0723	2.05	245.08 205.05 203.07 179.03 125.02 109.03	PVVE PVST PVBO
**38**	tuberonic acid hexoside isomer	10.59	C_18_H_28_O_9_	387.1662	3.36	207.10 163.11	All
**39**	eriodictyol-hexoside	11.16	C_21_H_22_O_11_	449.1097	4.23	259.06 287.06 269.05 179.00 125.02	PVVE PVST PVBO
**40**	quercetin xylopyranosyl-rutinoside	11.32	C_32_H_38_O_20_	741.1908	4.84	301.03 179.00	PVVE
**41**	quercetin sambubioside	11.84	C_26_H_28_O_16_	595.1306	2.21	301.03	PVVE
**42**	rutin	12.13	C_27_H_30_O_16_	609.1478	4.62	301.03	PVVE
**43**	quercetin hexoside	12.56–12.59–12.62	C_21_H_20_O_12_	463.0888	3.81	301.03	All
**44**	p-coumaric acid	12.83	C_9_H_8_O_3_	163.0395	3.37	119.05	All
**45**	quercetin malonylhexoside	12.96	C_24_H_22_O_15_	549.0899	4.50	301.03	All
**46**	quercetin acetylhexoside	13.10	C_23_H_22_O_13_	505.0999	4.52	301.03	All
**47**	sinapic acid	13.18	C_11_H_12_O_5_	223.0613	0.68	208.04 193.01 179.07 169.05 164.05 152.01 149.02	All
**48**	ferulic acid	13.33	C_10_H_10_O_4_	193.0503	4.37	178.03 134.04 149.06	All
**49**	kaempferol-hexoside	13.40	C_21_H_20_O_11_	447.0943	4.85	285.04	PVVE PVST PVBO
**50**	taxifolin	13.47	C_15_H_12_O_7_	303.0517	2.41	285.04 125.02	PVVE PVST PVBO PVCO
**51**	homovanillic acid	13.70	C_9_H_10_O_4_	181.0500	3.00	166.03	All
**52**	hesperetin	14.65	C_16_H_14_O_6_	301.0724	2.35	257.05	PVVE
**53**	quercetin	16.53	C_15_H_10_O_7_	301.0361	2.61	151.00 179.00	PVVE
**54**	α-hydroxyacetovanillone	17.40	C_9_H_10_O_4_	181.0500	3.00	166.03	All
**55**	soyasaponin V	18.72	C_48_H_78_O_19_	957.5073	2.12	457.37 221.07	All
**56**	soyasaponin I	19.07	C_48_H_78_O_18_	941.5122	1.94	457.37 205.07	All
**57**	sandosaponin (A/B)	19.90	C_48_H_76_O_19_	955.4939	4.38	455.35 221.07	All
**58**	dehydrosoyasaponin I	20.21	C_48_H_76_O_18_	939.49908	4.56	205.07	All
	*Positive ion mode*						
	**Compound**	**Rt (min)**	**Molecular Formula**	**[M + H]^+^**	**ppm**	**MS/MS**	**Detection**
**59**	delphinidin-hexoside	11.81	C_21_H_21_O_12_	465.1012	−3.33	303.05	PVVE
**60**	cyanidin-hexoside	12.47	C_21_H_21_O_11_	449.1069	−2.08	287.05	PVVE

The compounds were identified using the “*Compound Discoverer software*” platform and literature data, such as references [1,58,59,60].

**Table 5:** Inhibition percentage at 40 µg/mL of dichloromethane extracts from five varieties of *P. vulgaris* against the isolated COX-2 enzyme and IC_50_ values. Data are expressed as means ± SD, for n = 3.

Compound (40 µg/mL)	Inhibition Percentage ± SD	IC_50_ ± SD (µg/mL)
PVCO	47.5 ± 7.3	31.15 ± 2.16
PVBO	44.3 ± 2.6	42.29 ± 3.11
PVST	43.7 ± 5.4	43.92 ± 1.86
PVCA	38.1 ± 7.5	\
PVVE	19.6 ± 3.9	\
Celecoxib (known inhibitor)	85.2 ± 2.3	\

## Data Availability

The original contributions presented in the study are included in the article; further inquiries can be directed to the corresponding author.

## Supplementary Materials

The following supporting information can be downloaded at: https://www.mdpi.com/article/10.3390/biom14101336/s1. Figure S1. LC-MS profiles of lipophilic extracts in negative mode of Italian commercial *P. vulgaris* varieties: Vellutina (a), Borlotti (b), Stregoni (c), Controne (d), Cannellino (e). Figure S2. LC-MS profiles of lipophilic extracts in positive mode of Italian commercial *P. vulgaris* varieties: Vellutina (a), Borlotti (b), Stregoni (c), Controne (d), Cannellino (e). Figure S3. LC-MS profiles of hydrophilic extracts in negative mode of Italian commercial *P. vulgaris* varieties: Vellutina (a), Borlotti (b), Stregoni (c), Controne (d), Cannellino (e). Figure S4. LC-MS profiles of hydrophilic extracts in positive mode of Italian commercial *P. vulgaris* varieties: Vellutina (a), Borlotti (b), Stregoni (c), Controne (d), Cannellino (e). Figure S5. Score scatter plot of the untargeted principal component analysis (PCA) performed on the lipophilic extracts of five Italian commercial P. vulgaris varieties: Borlotti (PVBO) (green), Cannellino (PVCA) (blue), Controne (PVCO) (red), Stregoni (PVST) (yellow), Vellutina (PVVE) (light blue). Figure S6. (a) Variable importance in projection on component 1. (b) Variable importance in projection on component 2. Figure S7. (a) Bar chart showing the peak areas of polar lipids identified by LC-ESI-HRMS in negative mode. (b) Bar chart showing the peak areas of polar lipids identified by LC-ESI-HRMS in positive mode. Figure S8. Structures of main polar lipids identified (a,b) and their fragmentation (c,d,e) Figure S9. Concentration–response curves for the analysis of dichloromethane extracts from five variants of *Phaseolus vulgaris* against isolated COX-2 enzyme.
