# Supplementary material for: Chemical Profiling of Polar Lipids and the Polyphenolic Fraction of Commercial Italian Phaseolus Seeds by UHPLC-HRMS and Biological Evaluation
Source: Biomolecules. 2024 Oct 20;14(10):1336. doi: 10.3390/biom14101336 (PMC11505683; doi:10.3390/biom14101336)
Supplement: Supplementary file 1 [file biomolecules-14-01336-s001.zip › biomolecules-3225388-supplementary.pdf]

Supporting Information

Chemical Profiling of Polar Lipids and the Polyphenolic Fraction of Commercial Italian *Phaseolus* Seeds by UHPLC-HRMS and Biological Evaluation

Vadym Samukha [1], Francesca Fantasma [1], Gilda D'Urso [2], Ester Colarusso [2], Anna Schettino [3], Noemi Marigliano [3], Maria Giovanna Chini [1,*], Gabriella Saviano [1], Vincenzo De Felice [1], Gianluigi Lauro [2], Francesco Maione [3], Giuseppe Bifulco [2], Agostino Casapullo [2,*] and Maria Iorizzi [1,*]

[1] Department of Biosciences and Territory, University of Molise, Contrada Fonte Lappone, 86090 Isernia, Italy; v.samukha@studenti.unimol.it (V.S.); fantasma@unimol.it (F.F.); saviano@unimol.it (G.S.); defelice@unimol.it (V.D.F.)

[2] Department of Pharmacy, University of Salerno, Via Giovanni Paolo II 132, 84084 Fisciano, Italy; gidurso@unisa.it (G.D.); ecolarusso@unisa.it (E.C.); glauro@unisa.it (G.L.); bifulco@unisa.it (G.B.)

[3] ImmunoPharmaLab, Department of Pharmacy, School of Medicine and Surgery, University of Naples Federico II, Via Domenico Montesano 49, 80131 Naples, Italy; anna.schettino2@unina.it (A.S.); noemi.marigliano@outlook.com (N.M.); francesco.maione@unina.it (F.M.)

* Correspondence: mariagiovanna.chini@unimol.it (M.G.C.); casapullo@unisa.it (A.C.); iorizzi@unimol.it (M.I.); Tel.: +39-0874404132 (M.G.C.); +39-089969243 (A.C.); +39-0874404100 (M.I.)

Table of contents

Figure S1. LC-MS profiles of lipophilic extracts in negative mode of commercial Italian *P. vulgaris* varieties: Vellutina (a), Borlotti (b), Stregoni (c), Controne (d), Cannellino (e)................................. 3

Figure S2. LC-MS profiles of lipophilic extracts in positive mode of commercial Italian *P. vulgaris* varieties: Vellutina (a), Borlotti (b), Stregoni (c), Controne (d), Cannellino (e)................................. 4

Figure S3. LC-MS profiles of hydrophilic extracts in negative mode of commercial Italian *P. vulgaris* varieties: Vellutina (a), Borlotti (b), Stregoni (c), Controne (d), Cannellino (e)..................... 5

Figure S4. LC-MS profiles of hydrophilic extracts in positive mode of commercial Italian *P. vulgaris* varieties: Vellutina (a), Borlotti (b), Stregoni (c), Controne (d), Cannellino (e)................................. 6

Figure S5. Score Scatter Plot of the Untargeted Principal Component Analysis (PCA) performed on the lipophilic extracts of five commercial Italian *P. vulgaris* varieties: Borlotti (PVBO) (green), Cannellino (PVCA) (blue), Controne (PVCO) (red), Stregoni (PVST) (yellow), Vellutina (PVVE) (light blue). 7

Figure S6. a) Variable Importance in Projection on component 1. **b)** Variable Importance in Projection on component 2.. 7

Figure S7. a) Bar chart showing the peak areas of polar lipids identified through LC-ESI-HRMS in negative mode. **b)** Bar chart showing the peak areas of polar lipids identified through LC-ESI-HRMS in positive mode. 8

Figure S8. Structures of main polar lipids identified (**a; b**) and their fragmentation (**c; d; e**)........... 9

Figure S9. Concentration-response curves for the analysis of dichloromethane extracts from five variants of *Phaseolus vulgaris* against isolated COX-2 enzyme. Data are expressed as percentage of control (100%), means with SD, n = 3. 10

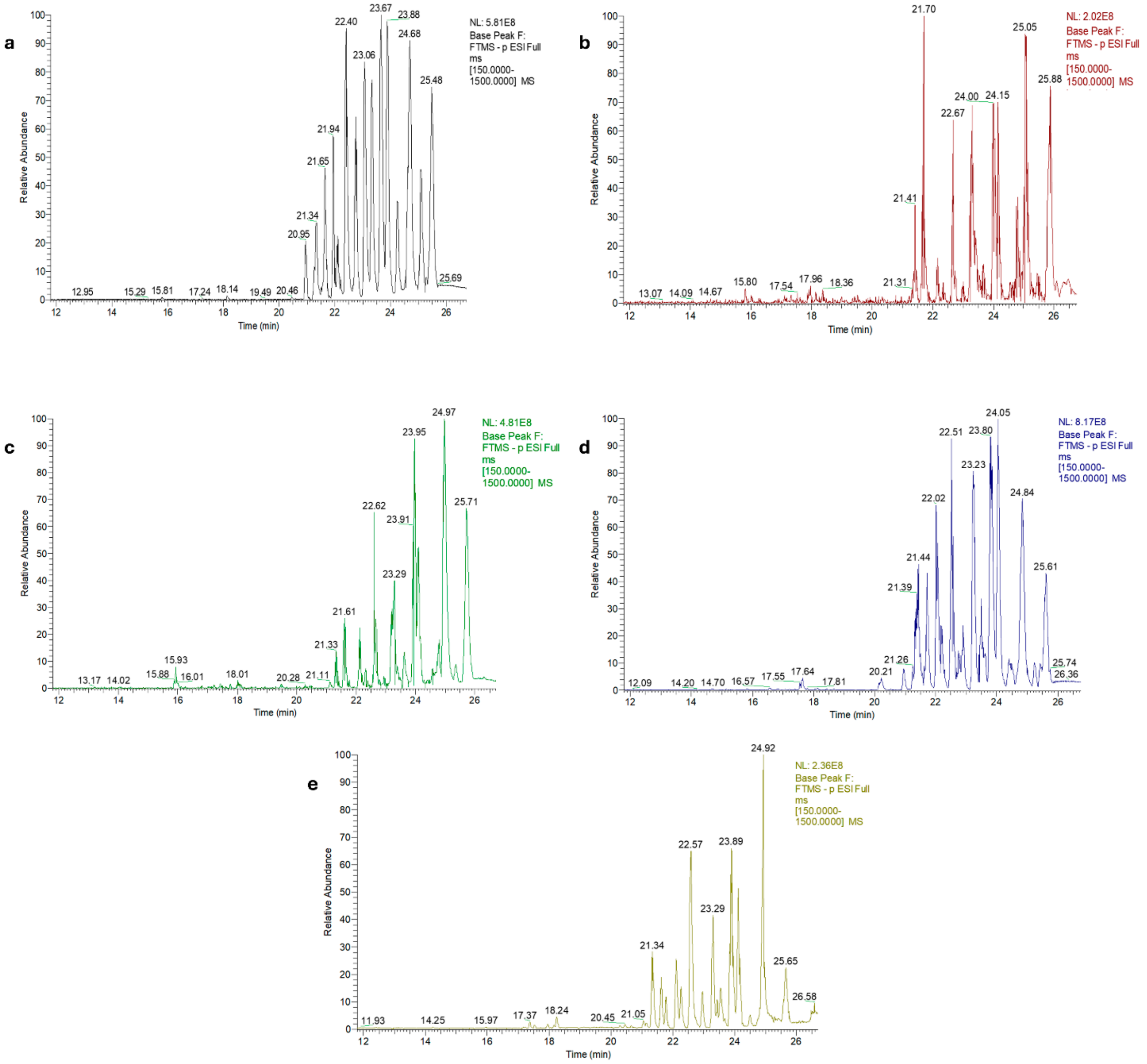

Figure S1. LC-MS profiles of lipophilic extracts in negative mode of commercial Italian *P. vulgaris* varieties: Vellutina (a), Borlotti (b), Stregoni (c), Controne (d), Cannellino (e).

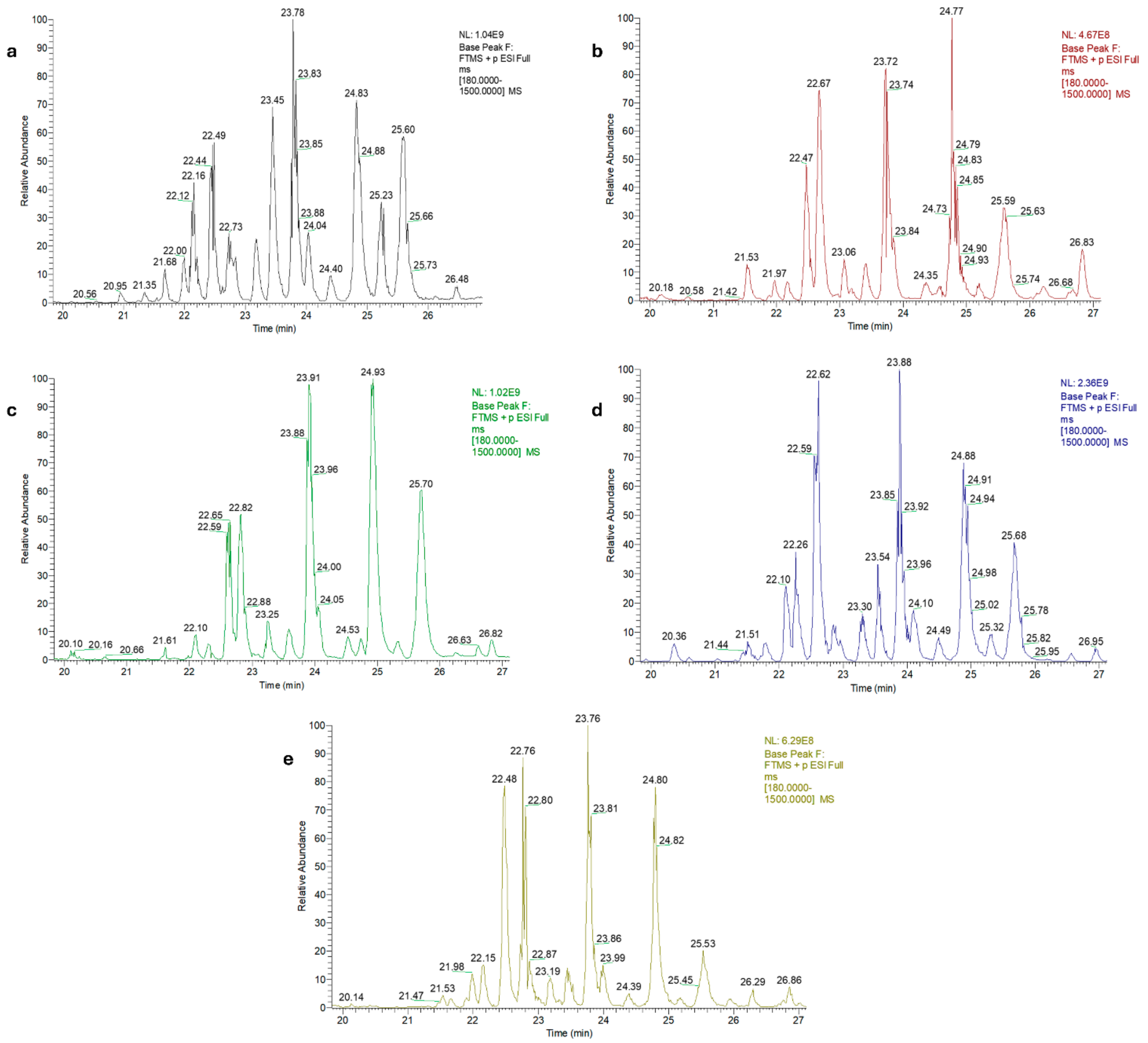

Figure S2. LC-MS profiles of lipophilic extracts in positive mode of commercial Italian *P. vulgaris* varieties: Vellutina (a), Borlotti (b), Stregoni (c), Controne (d), Cannellino (e).

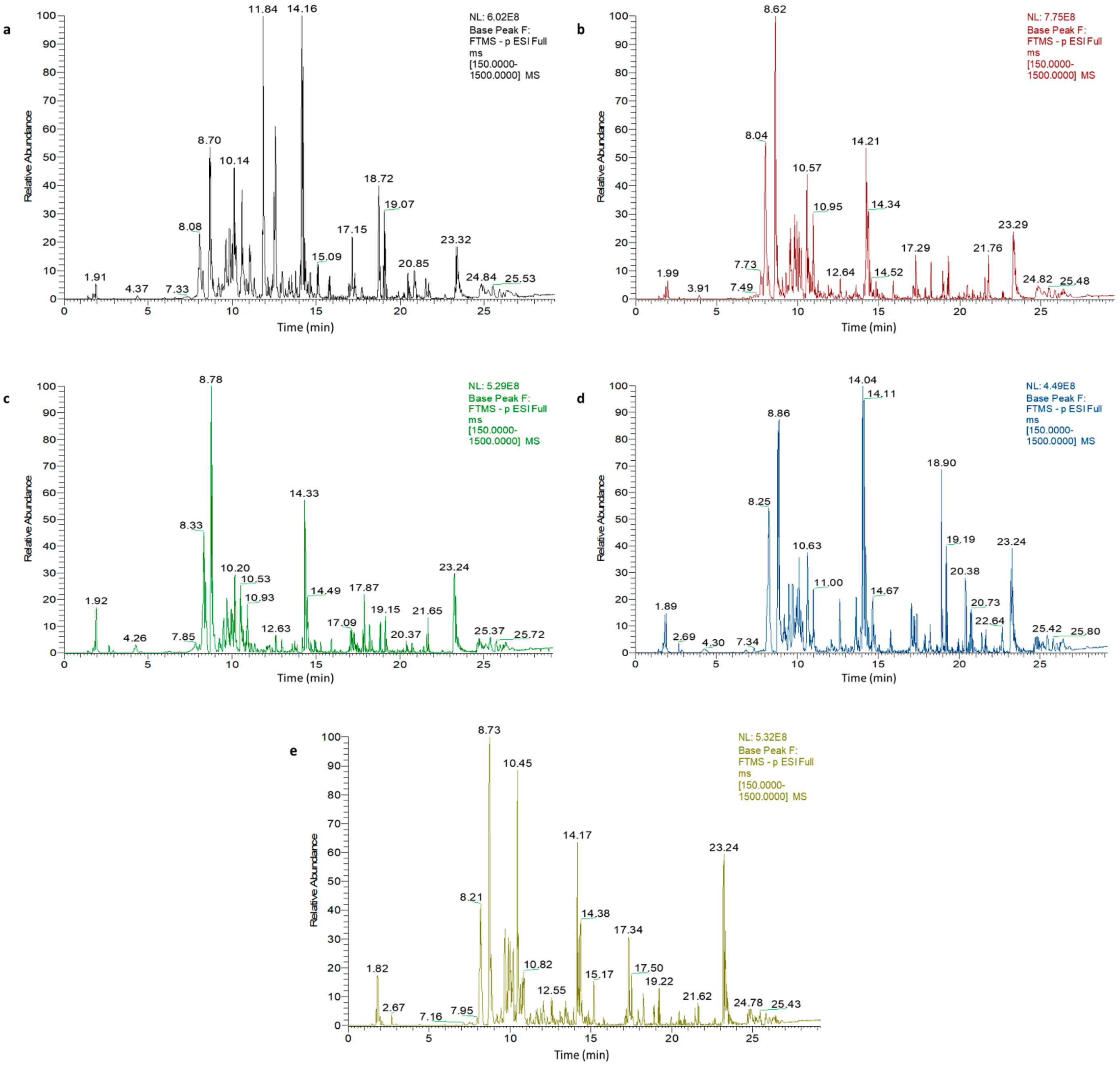

Figure S3. LC-MS profiles of hydrophilic extracts in negative mode of commercial Italian *P. vulgaris* varieties: Vellutina (a), Borlotti (b), Stregoni (c), Controne (d), Cannellino (e).

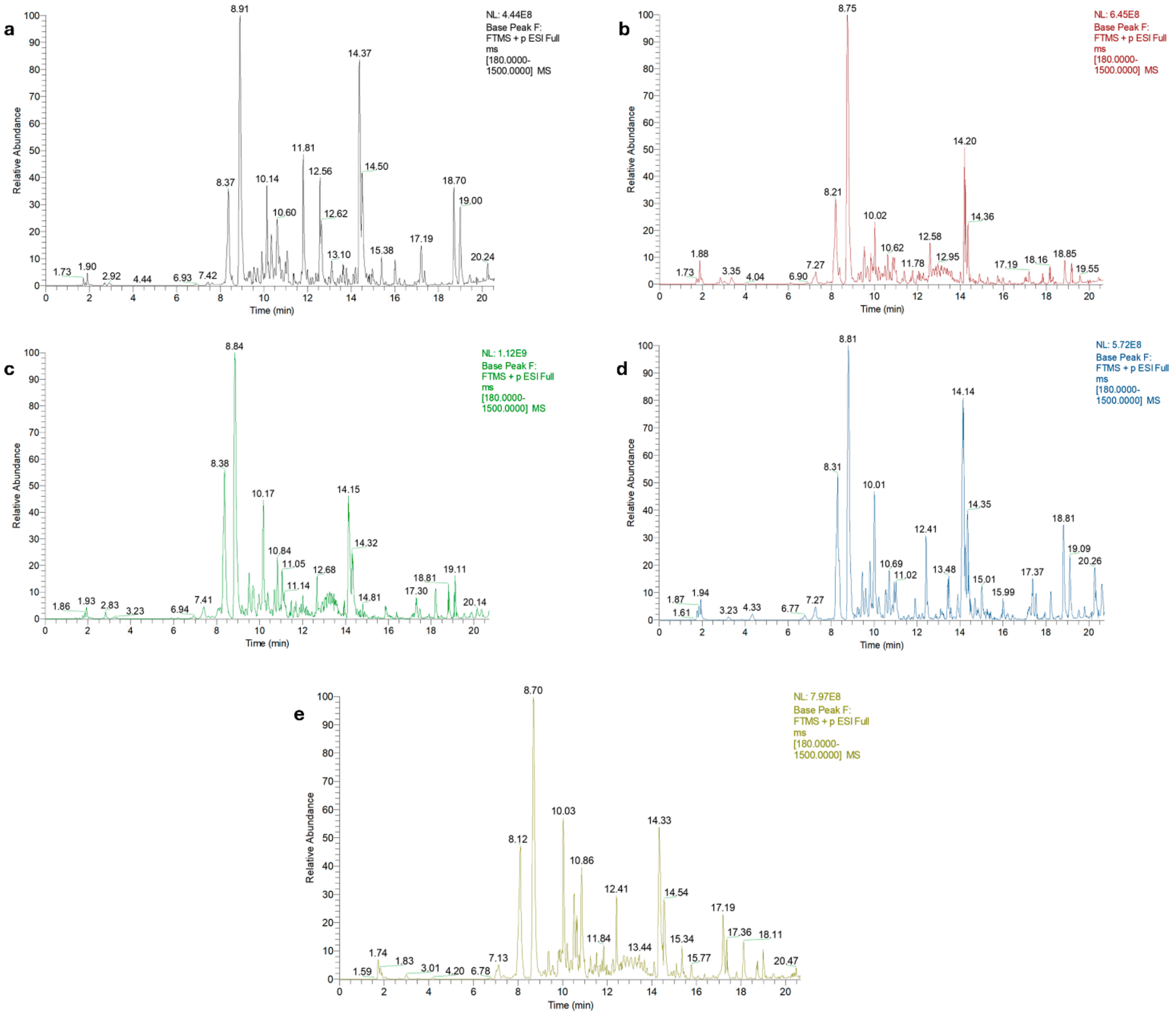

Figure S4. LC-MS profiles of hydrophilic extracts in positive mode of commercial Italian *P. vulgaris* varieties: Vellutina (a), Borlotti (b), Stregoni (c), Controne (d), Cannellino (e).

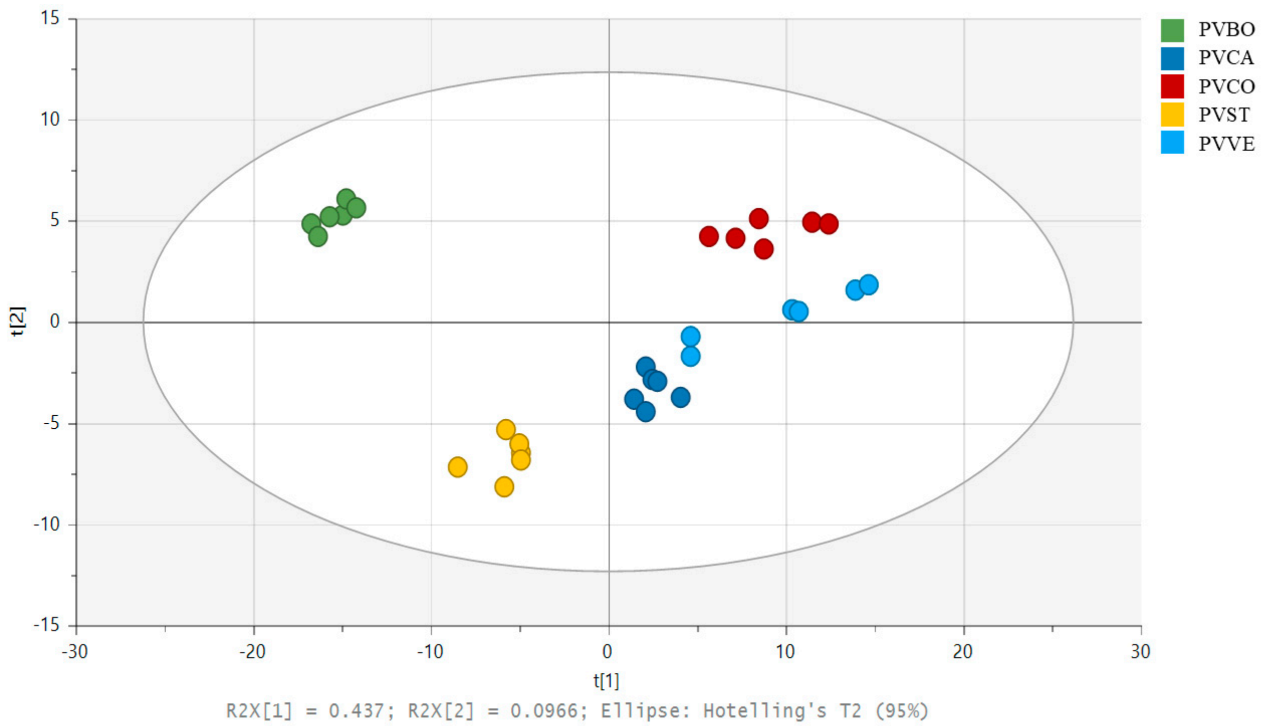

Figure S5. Score Scatter Plot of the Untargeted Principal Component Analysis (PCA) performed on the lipophilic extracts of five Italian commercial *P. vulgaris* varieties: Borlotti (PVBO) (green), Cannellino (PVCA) (blue), Controne (PVCO) (red), Stregoni (PVST) (yellow), Vellutina (PVVE) (light blue).

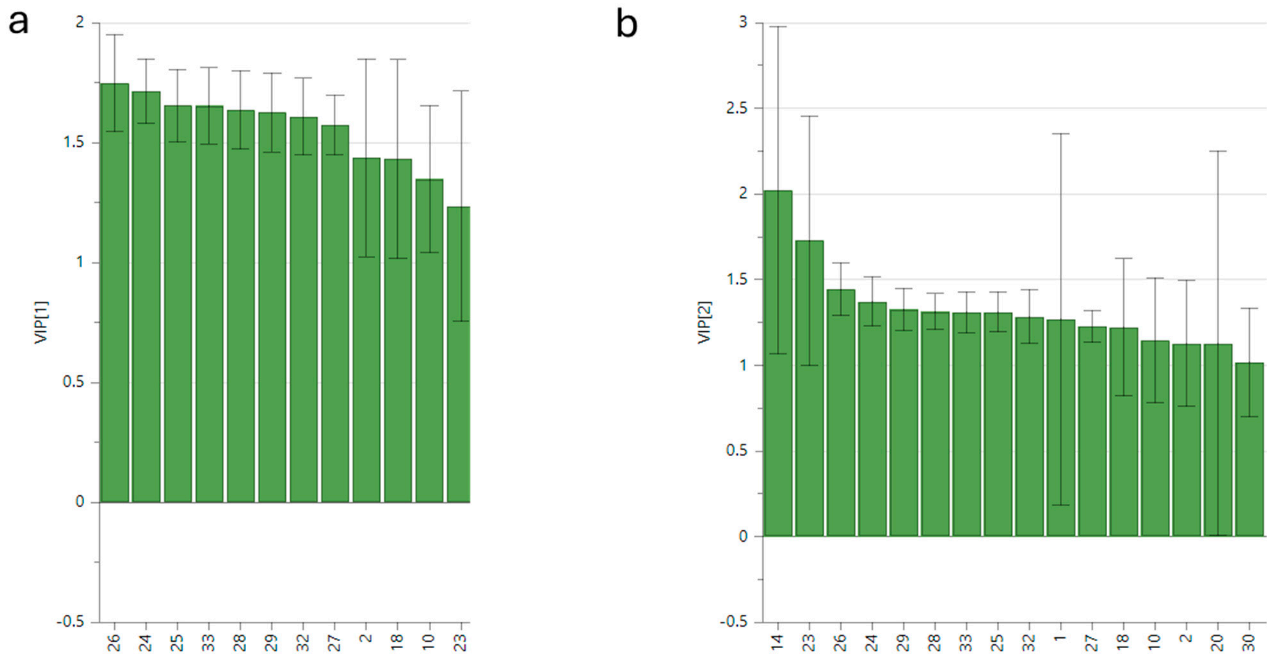

Figure S6. a) Variable Importance in Projection on component 1. **b)** Variable Importance in Projection on component 2.

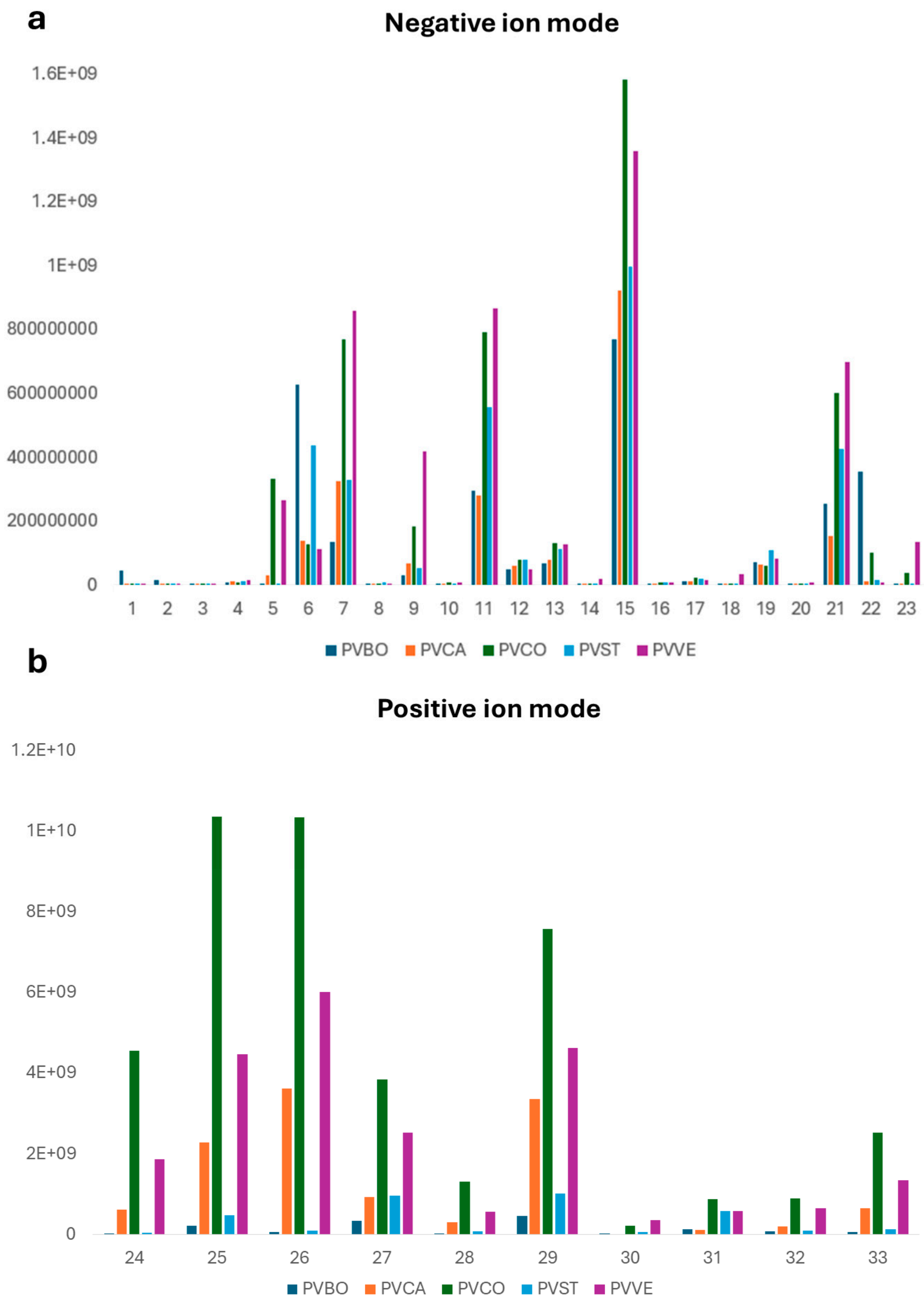

Figure S7. a) Bar chart showing the peak areas of polar lipids identified through LC-ESI-HRMS in negative mode. **b)** Bar chart showing the peak areas of polar lipids identified through LC-ESI-HRMS in positive mode.

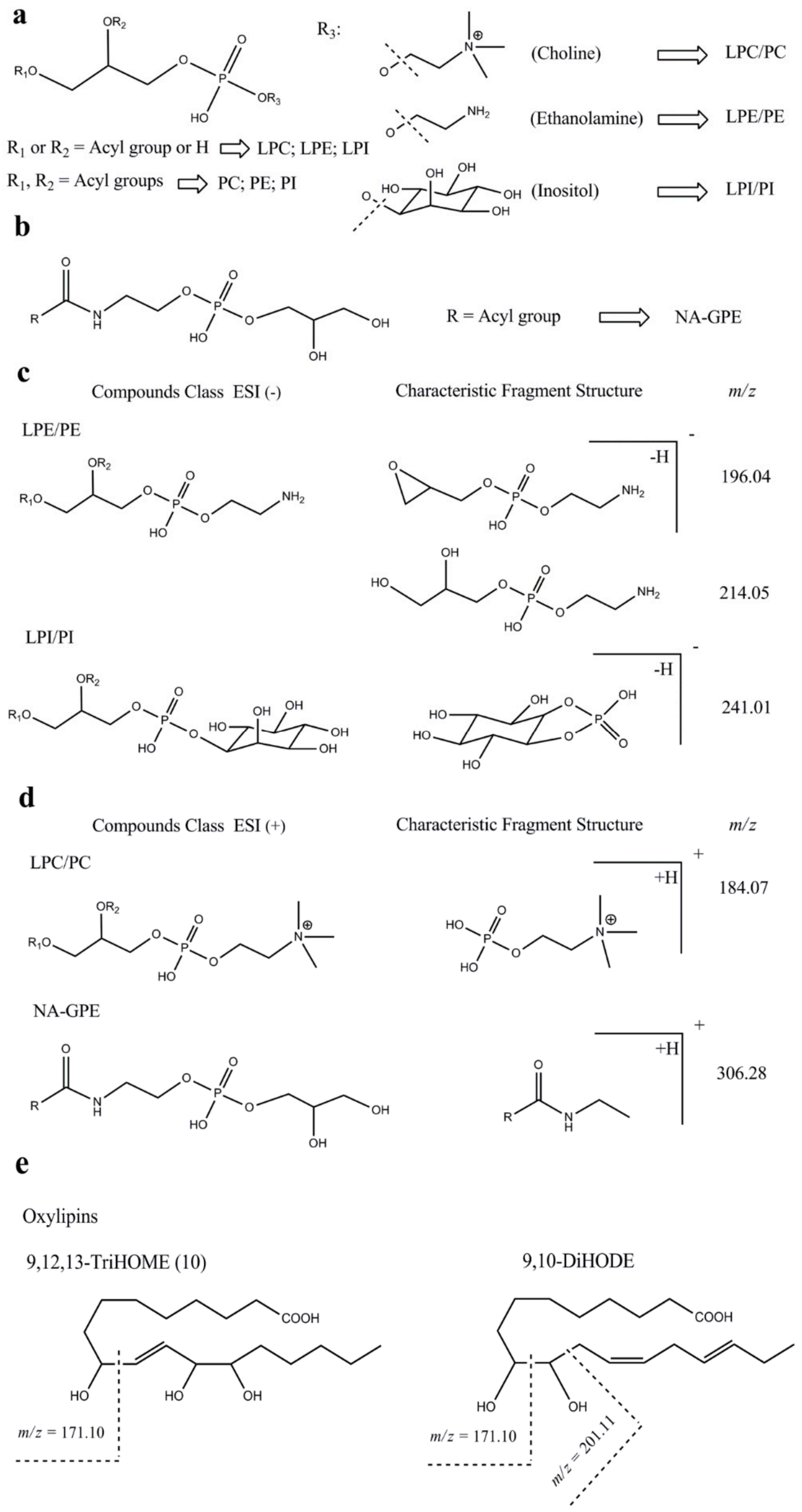

Figure S8. Structures of main polar lipids identified (**a; b**) and their fragmentation (**c; d; e**).

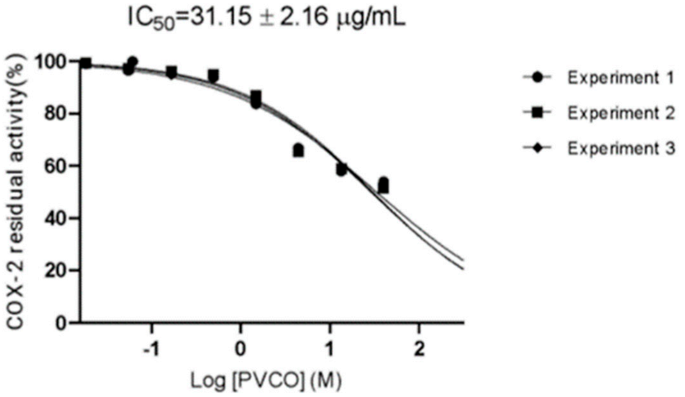

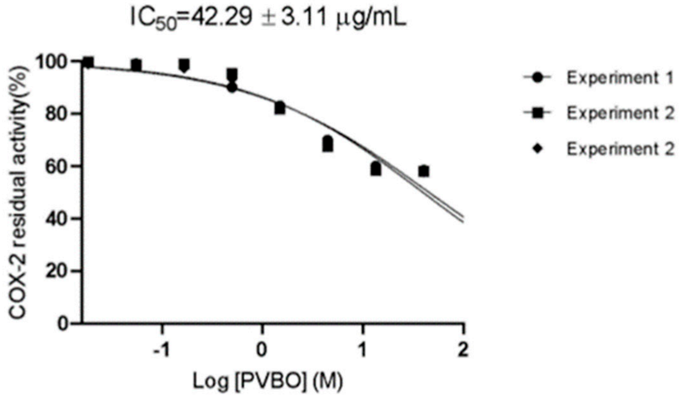

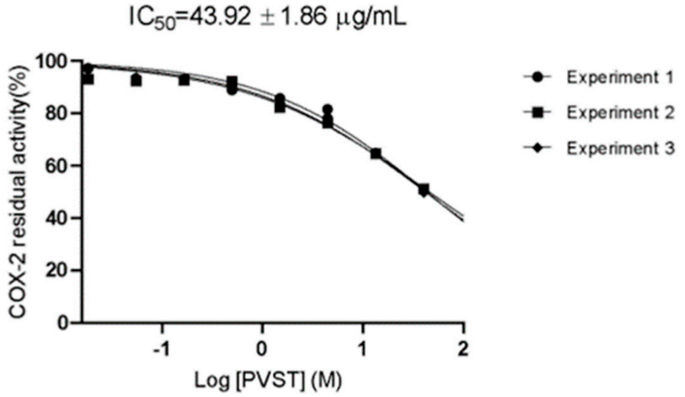

Figure S9. Concentration-response curves for the analysis of dichloromethane extracts from five variants of *Phaseolus vulgaris* against isolated COX-2 enzyme. Data are expressed as percentage of control (100%), means with SD, n = 3.
